# Different clinical manifestations of ocular sporotrichosis in the same patient: an alert to ophthalmologists in nonendemic areas

**DOI:** 10.5935/0004-2749.20200107

**Published:** 2024-02-11

**Authors:** Alexandre de Carvalho Mendes Paiva, Ana Luiza Biancardi, André Luiz Land Curi

**Affiliations:** 1 Ophthalmology Department, Universidade Federal do Estado do Rio de Janeiro, Rio de Janeiro, RJ, Brazil; 2 Instituto Nacional de Infectologia, Fundação Oswaldo Cruz, Rio de Janeiro, RJ, Brazil

To the editor:

Human and animal sporotrichosis is a subacute or chronic infection caused by thermodimorphic fungi belonging to the genus *Sporothrix*. It has a cosmopolitan distribution; however, there are notably endemic areas in Latin America, such as Rio de Janeiro, Brazil. This infection is classically transmitted by traumatic subcutaneous inoculation of contaminated materials such as soil, plant, or organic matter, generally associated with occupational activities such as gardening and agricul ture^(1)^. In Rio de Janeiro, the zoonotic mode of trans mission, through bites, scratch, or contact with secretions of infected cats, is more common^(2)^.

Currently, sporotrichosis is classified in four types. The lymphocutaneous form (80% of the cases) is characterized by nodular or ulcerative lesions in the inoculation site and progresses along the local lymphangitic channels. The classic cutaneous form manifests as a single lesion at the site of inoculation ^(1)^. The extracutaneous form is uncommon and includes pulmonar, osteoarticular, ocular, or central nervous system disorders^(3)^. The disseminated form of sporotrichosis occurs in immunocompromised patients^(1,3)^.

Ocular sporotrichosis occurs due to trauma, autoinoculation, or hematogenous dissemination. Granulomatous conjunctivitis is the commonest presentation. The simultaneous occurrence of conjunctivitis and preauricular or submandibular lymph nodes is defined as Parinaud’s oculoglandular syndrome^(2)^.

**Figure 1:**
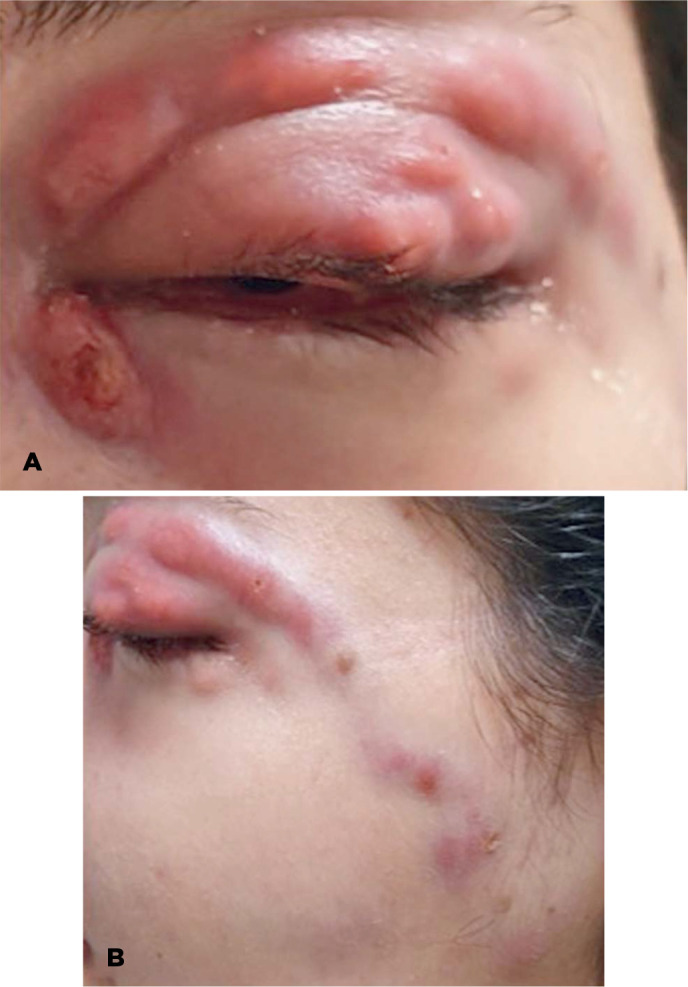
Lymphocutaneous and ocular sporotrichosis. A) Upper eyelid edema and nodular lesions. B) Progression of the lesions to regional lymphatic channels and preauricular lymph node enlargement.

**Figure 2:**
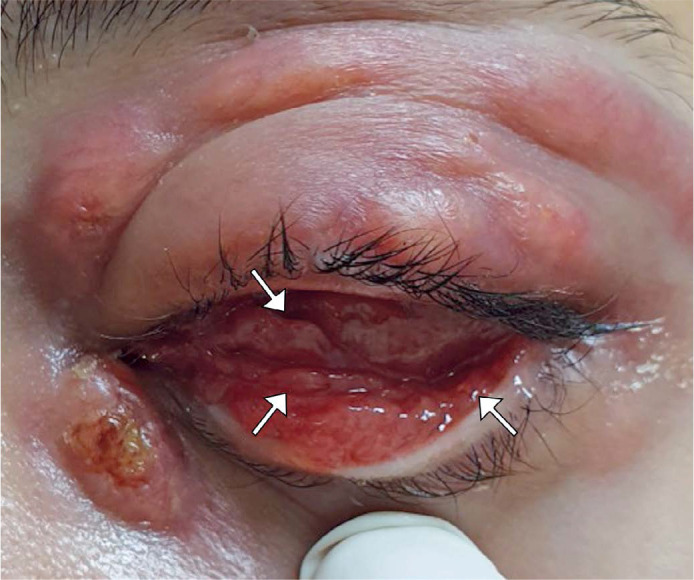
Bulbar and lower tarsal granulomatous conjunctivitis (arrows) with fistulizing dacryocystitis.

Herein, we describe a case of a healthy 25-year-old woman who had complaints of ocular hyperemia, mucopurulent discharge, and foreign body sensation in the left eye (OS). Her past ocular history was unremarkable. She stated that her domestic cat had sporotrichosis that was diagnosed before the initiation of ocular symptoms. Ocular examination of the OS disclosed upper eyelid edema and nodular lesions that progressed to regional lymphatic channels (Figure 1A), bulbar and lower tarsal granulomatous conjunctivitis, and fistulizing dacryocystitis (Figure 2). Visual acuity, tonometry, and fundoscopy revealed unremarkable findings. Ophthalmic examination of the right eye showed normal findings. Left preauricular and submandibular lymph node enlargement was also observed (Figure 1B). The patient underwent mycological examination of the ocular secretion, in which the culture was positive to *Sporothrix* spp. She was treated with itraconazole 200 mg/day orally for 3 months with improvement, but the fistula remained open.

Increased rates of human sporotrichosis caused by *S. brasiliensis* with the zoonotic mode of transmission have been reported in Brazil. The primary referral center for this disease, The Oswaldo Cruz Foundation (Fiocruz), in Rio de Janeiro, had registered 5000 cases of human disease during 1998-2015 and 5113 cases of feline disease during 1998-2018^(4)^. In 2019, until the month of December, 242 suspected cases were reported to the health department of Rio de Janeiro, of which 214 (88.4%) were confirmed^(5)^.

A geographic expansion of sporotrichosis has been detected over the past 20 years. The southeastern region has the highest number of cases of human and animal sporotrichosis. In regions where only feline cases are reported, the zoonotic form may be underreported and neglected. Today, there are cases of both human and animal disease in the southeastern regions Rio Grande do Sul and Alagoas. However, cases of feline sporotrichosis, without notifications of human disease, have been reported in Santa Catarina, Paraná, Mato Grosso, and Pernambuco e Rio Grande do Norte. In the states of Paraíba, Mato Grosso do Sul, Pará, and Acre, there were cases of human and feline disease reported from other sources of communication, such as electronic media^(4)^.

The present case indicates a warning to Brazilian ophthalmologists who are unfamiliar with sporotrichosis. It describes the different clinical manifestations of ocular sporotrichosis in the same patient, i.e., eyelid nodular lesions with progression through lymphangitic channels, Parinaud’s oculoglandular syndrome, and dacryocystitis with fistula.

As the epidemic of sporotrichosis has already expanded from Rio de Janeiro to the other states of the southeastern region, and progressively to other Brazilian states, there is a need for a high degree of suspicion in these cases. Epidemiological investigation must inquire aspects such as contact with cats, especially those with dermatological diseases, and recent trips to endemic areas.
